# Teaching an old vector new tricks: the surprising versatility of AAV vaccines

**DOI:** 10.1128/jvi.00730-25

**Published:** 2025-07-14

**Authors:** Stephen M. Winston, Kristin B. Wiggins, Stacey Schultz-Cherry, Andrew M. Davidoff

**Affiliations:** 1 Department of Surgery, St. Jude Children's Research Hospital 5417, Memphis, Tennessee, USA; 2 Department of Host–Microbe Interactions, St. Jude Children's Research Hospital 5417, Memphis, Tennessee, USA; Indiana University Bloomington, Bloomington, Indiana, USA

**Keywords:** AAV, vaccine

## Abstract

Adeno-associated virus (AAV) has proven its clinical efficacy in the realm of gene therapy, resulting in seven FDA-approved gene therapies. While AAV gene transfer research has predominantly focused on its utility in monogenic disorders, AAV vectors have been used as a platform for vaccines in over 50 preclinical studies over the last 25 years. Recombinant AAV-based vaccines have demonstrated induction and durability of antigen-specific adaptive immune responses in a variety of preclinical models. This mini-review serves as a comprehensive discussion of the basics of vaccine vector design and experimental considerations, highlighting engineering efforts to improve AAV vaccine efficacy, along with the known advantages and disadvantages of AAV-based vaccines from published pre-clinical studies.

## INTRODUCTION

The most common FDA-approved vaccines against viral pathogens use inactivated or attenuated whole viruses. An alternative approach uses recombinant viral vectors to deliver genetic material encoding an antigen to the host cells to produce that antigen within the cell to trigger an immune response. Some examples of viral vectors used in a vaccine context include modified vaccinia virus Ankara, raccoonpox virus, herpesviruses, and baculovirus; however, the most frequently used are adenovirus vectors (1). There have been successful clinical trials of recombinant, replication-incompetent human adenovirus type 26 (Ad26) vectors as vaccine platforms against numerous viruses, including severe acute respiratory syndrome coronavirus 2 (SARS-CoV-2), Zika virus, filovirus, human immunodeficiency virus (HIV), and respiratory syncytial virus (RSV), that safely generated immune responses sufficient for protection against severe disease (2–10). Adenoviral vector vaccines introduced during the SARS-CoV-2 pandemic showed promise in terms of their production value, scalability, immunogenicity, and efficacy from a single dose, with repeat administration boosting antibody responses up to ninefold (2, 11–13). Unfortunately, rare but serious links with transient thrombosis in individuals with underlying medical conditions revealed that more research was warranted into vaccine responses across all populations, and its emergency use authorization was revoked (2, 11, 14). Though not from a vaccine study, the unintended risks of using adenoviral vectors are highlighted in the story of Jesse Gelsinger, who died from complications of vector administration in a gene therapy trial for ornithine transcarbamoylase deficiency.

**Fig 1:**
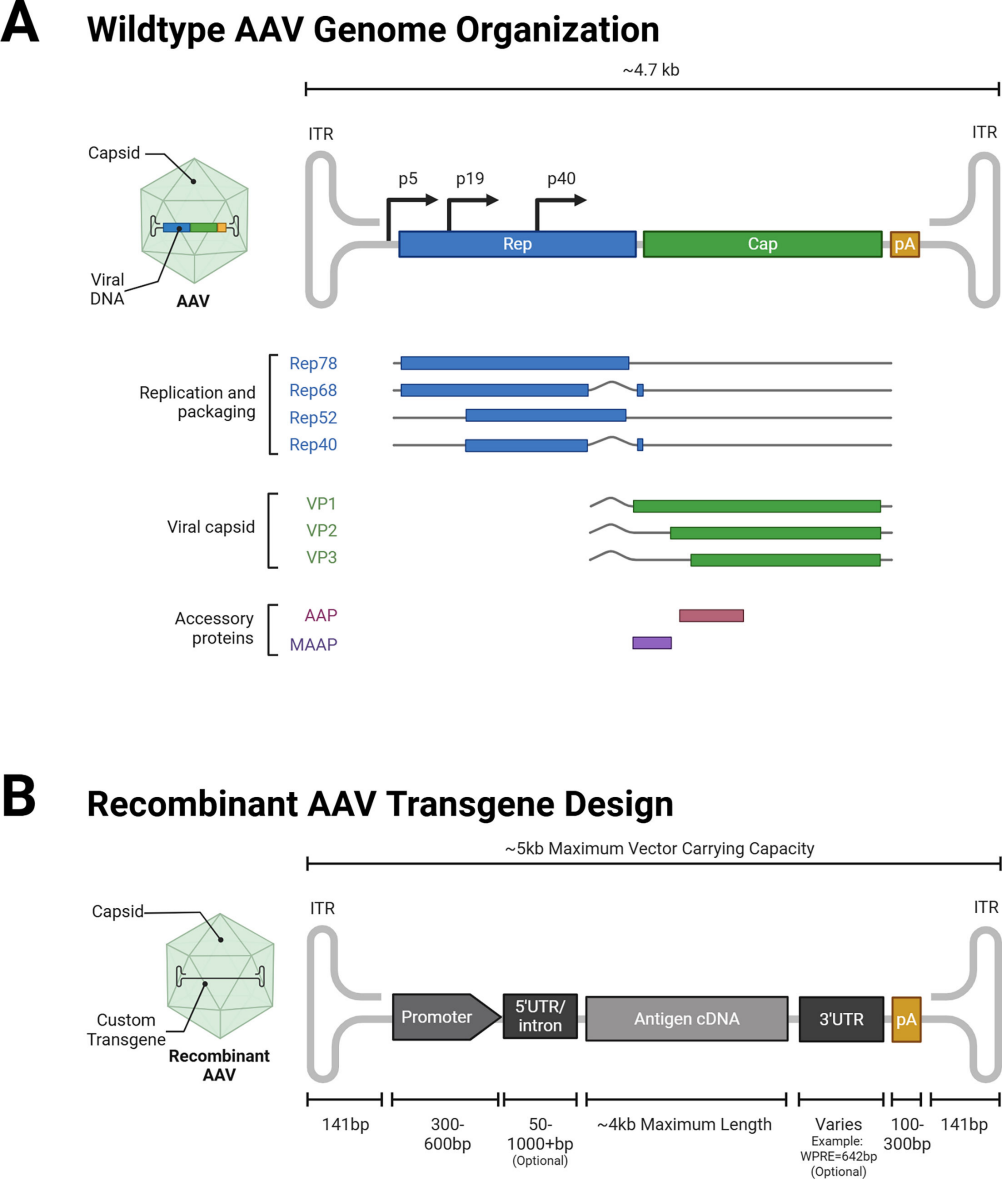
AAV vector design. (**A**) Organization of the wild-type AAV genome, highlighting the replication/packaging, viral capsid, and accessory protein genes. (**B**) Recombinant AAV transgene design, showing the standard structure for vaccine design.

Better known for its use in gene therapy than as a vaccine platform is adeno-associated virus (AAV). Initially described as a contaminant of adenovirus and later other viruses, in culture (15–17), AAV proved to be a distinct entity reliant on these other viruses for replication. Multiple serotypes and AAV-specific proteins have since been identified. The compact AAV genome consists of inverted terminal repeats (ITRs) flanking the two main reading frames for the replication and capsid-forming genes. These reading frames use embedded promoters and translation start sites, and differential splicing to encode 11 proteins (18) (Fig. 1A). After extensive characterization of the helper genes required for replication, AAV became a valuable tool for efficient viral recombination and is now considered an effective and safe vector for gene transfer (18–25). AAV has also shown promise as a tool for monoclonal antibody delivery for a variety of diseases, notably HIV (26–30).

**Fig 2:**
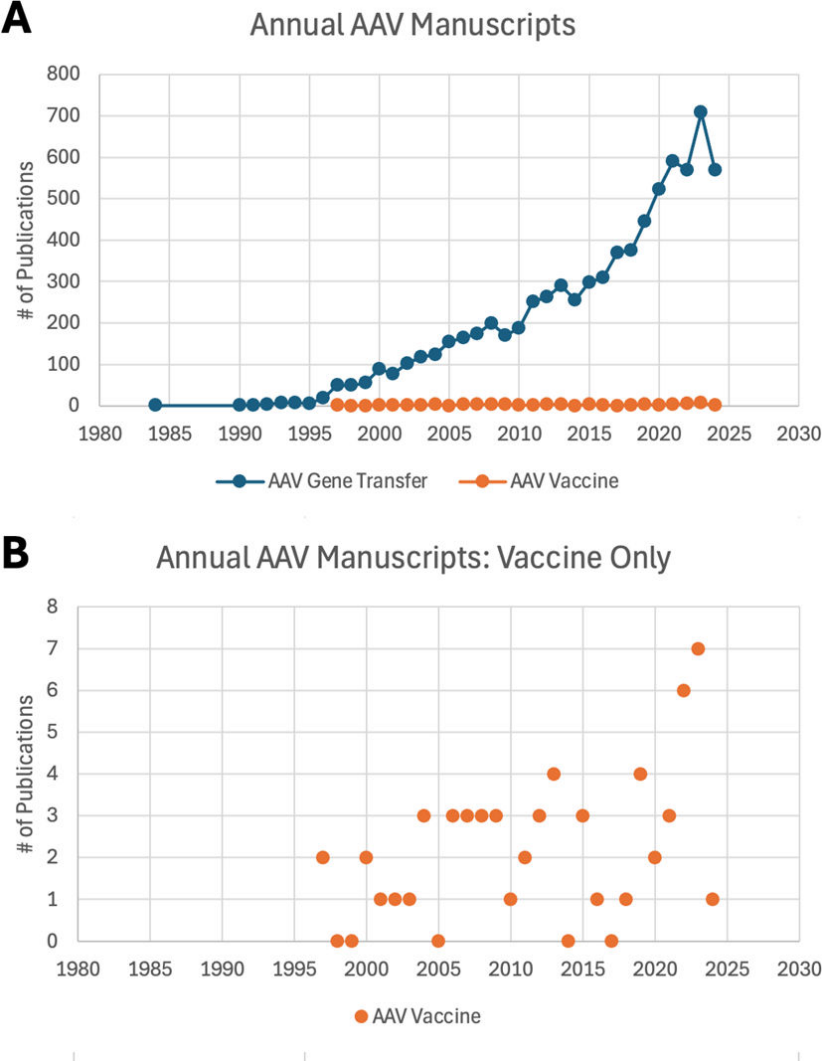
AAV publications focused on gene therapy versus vaccines. (**A**) Number of AAV-related publications indexed in PubMed each year that were focused on gene transfer versus vaccines, highlighting the paucity of research into AAV as a vaccine tool. (**B**) Same annual data for vaccine-focused publications as in (**A**), but with the y-axis scale adjusted to show the exact number per year.

Although hundreds of papers describing the use of AAV in gene replacement therapy are published each year, there have been far fewer published studies of its utility as a vaccine platform (Fig. 2). Despite its relatively low immunogenic profile, AAV can be used for this purpose by activating multiple components of the innate and adaptive immune responses, resulting in durable protection in multiple animal models.

## MANIPULATING AAV AS A VACCINE PLATFORM

There are many ways to modulate AAV vectors, as the genome and capsid are highly permissive for manipulation (Fig. 1B). The vector genomes may be self-complementary (scAAVs) or single-stranded. scAAV vectors use a terminal resolution site-deleted ITR to enable packaging of the complementary DNA strand. scAAV vectors have smaller carrying capacities, but because they bypass the need for second-strand synthesis, they can become transcriptionally active immediately after infecting and then folding into double-stranded DNA in the transduced host cell to enable efficient protein synthesis (18).

**Fig 3:**
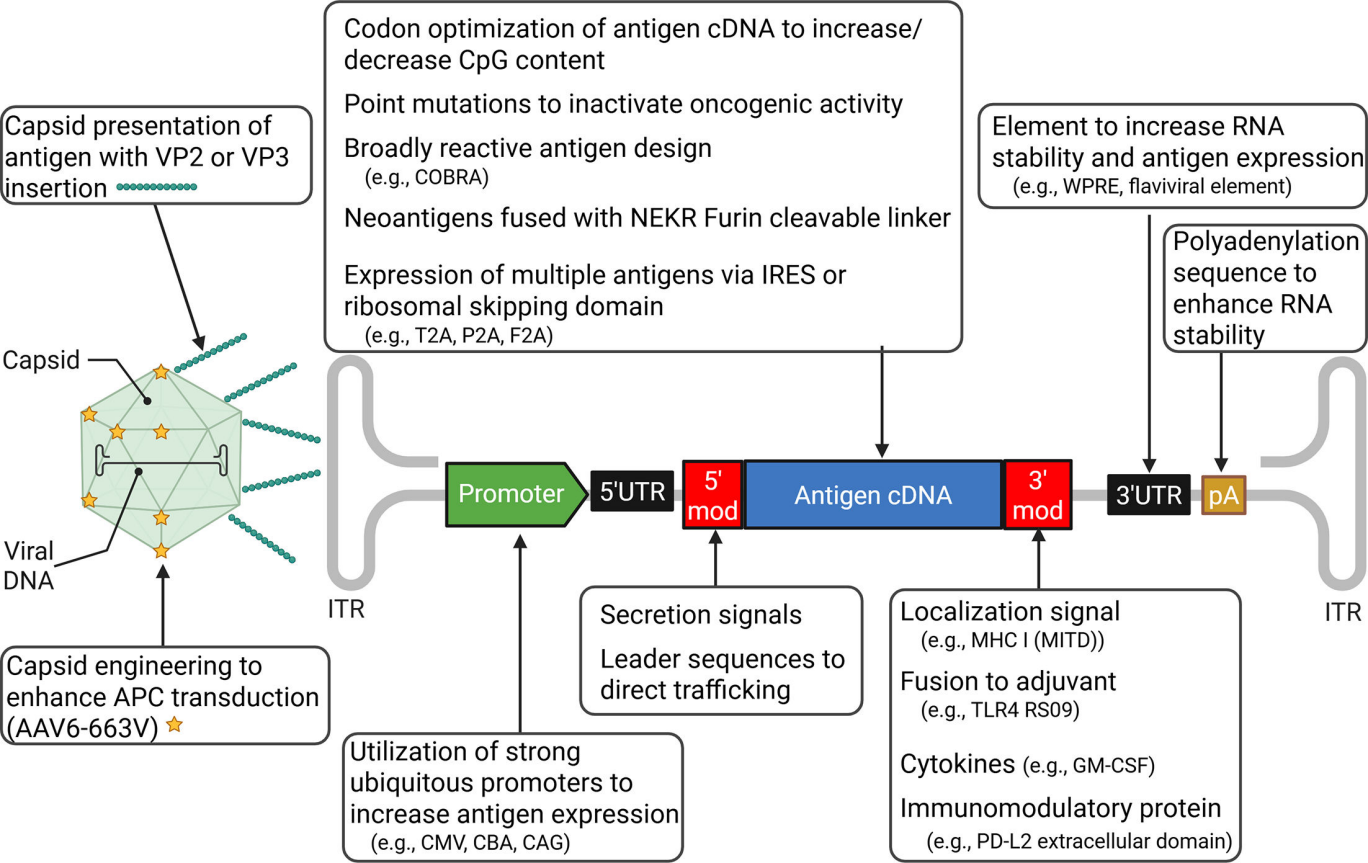
AAV vector modifications for enhanced vaccine efficacy. Numerous AAV vector modifications can be introduced to improve vaccine efficacy. The modifications span sites throughout the vector, including changes to the capsid, the antigen, or the promoter itself.

Recombinant AAV genomes predominantly use AAV serotype-2 ITRs, which can be cross-packaged into any natural or synthetic capsid serotype. Capsid serotypes are responsible for the tissue tropism of the vector (Fig. 1B). Although most capsids are highly hepatotropic, some naturally occurring and synthetic capsids have high muscle tropism (e.g., AAV9 and AAVMYO), lung tropism (e.g., AAV4 and AAV2.5), or leukocyte tropism (e.g., AAV6 and ARK313) (31–38). This tropism can be exploited to target particular sites, like using AAV5 or AAV6 to target dendritic cells to theoretically boost vaccine responses (39). There were minimal differences in efficacy in head-to-head capsid comparisons (40). Not only can the selected capsid dictate vector tropism and transduction efficiency, but it can also influence the durability of transgene expression and immune cell infiltration following vector administration; the mechanisms behind this are not entirely understood (41, 42). Immune responses to the capsid are extensively reviewed elsewhere (43–45). AAV capsids are also permissive for insertions at various locations; this property was initially exploited to add fluorescent reporters to the capsids to track them during transduction. This strategy has since become a popular means of capsid engineering to redirect vector tropism (46–50). Preclinical studies of vaccines for infectious disease and cancer models have used this strategy to present immunodominant antigens on the capsid surface (Fig. 3); some mouse studies have demonstrated vaccine efficacy when using antigenic capsid insertions even without a transgene (51–53).

Different promoters and regulatory elements can also be used to control AAV vector expression (Fig. 1B). Cytomegalovirus (CMV), CMV early enhancer/chicken β-actin (CAG), and chicken β-actin (CBA) promoters are commonly used to generate strong expression of the encoded transgene (Fig. 3). Additional transgene features can include introns, untranslated regions, the woodchuck hepatitis virus post-transcriptional regulatory element, and a polyadenylation sequence (PolyA), each of which enhances RNA stability and nuclear export, resulting in improved transgene expression (54, 55) (Fig. 3). Combining strong promoters with other elements to increase antigen expression can further augment vector efficacy.

The efficacy of AAV treatment for monogenic disorders depends on vector genome persistence and lasting transgene expression. Although durable expression is routinely observed in AAV gene transfer when the vector encodes an endogenous protein, delivering foreign proteins often results in the clearance of transduced cells; this can sometimes be alleviated using cell-type-specific promoters or other means of preventing transgene expression in immune cells (56). In one study, gene editing accomplished by AAV-mediated delivery of Cas9 was negated by the clearance of the transduced cells (57). Persistent Cas9 expression in edited hepatocytes triggered an adaptive immune response. Over time, there was an inverse correlation between the percentage of edited cells and that of Cas9-reactive CD8+ T cells. Furthermore, in our study of an AAV influenza vaccine in mice, quantitative PCR analysis of genomic DNA from the injected muscle 6 weeks post-vaccination revealed little vector genome persistence, regardless of the capsid used (40). Similarly, in a preclinical study of a vaccine against Nipah virus, analysis of the injected mouse muscle showed a significant decline in vector genome persistence over time (58). Such limited persistence has also been observed in larger animal models like rhesus macaques (59).

Vector design modifications to enhance antigen presentation to dendritic cells can influence genome persistence (60). For example, using the MHC leader peptide and the C-terminal MHC class I trafficking signal (MITD) to promote antigen presentation and immune detection enhanced vector genome clearance. When using luciferase tracking in mice, adding these elements resulted in bioluminescence being significantly reduced by day 11 and declining to background levels 17 days post-injection, whereas the unmodified luciferase vector largely maintained expression through day 17.

On the other hand, other groups have used immune-suppressive features to prolong vector genome persistence and antigen expression in mice (61). Although this approach enhances the efficacy of anti-cancer vaccines, prolonged expression of HIV antigens by AAV vectors is believed to be associated with T-cell dysfunction (62). The timing of antigen expression may require optimization for the specific target; some vaccines may require durable expression, whereas others may require transient expression. CpG depletion of the expression cassette can also minimize the immunogenicity of the vector, increasing genome persistence and prolonging antigen expression. AAV genomes contain predominantly unmethylated CpGs, which activate TLR9 and lead to transaminitis and subsequent decreased gene expression in human clinical trials (63–65). To inhibit TLR9, efforts have been made to fuse the therapeutic antigen to TLR9-inhibitory peptides (61). Alternative TLR9-inhibiting nucleic acid sequences have been validated in AAV vectors to augment this strategy further (66) (Fig. 3). Without any head-to-head comparisons, it is unclear whether decreased or increased immunogenicity is more effective for AAV vaccines.

Rather than minimizing the vector immunogenicity to prolong expression, some groups have used adjuvanting features to augment the initial immune response after vaccination. CpG enrichment and TLR9 activation can enhance the adaptive immune response by the host (63). We have shown how CpG enrichment in the antigen-coding sequence of an AAV influenza vaccine serves as an internal adjuvant. Codon optimization of the influenza hemagglutinin H1 antigen enabled the incorporation of >300 CpG motifs. The CpG-enriched vectors enabled dose de-escalation while maintaining 100% survival after challenge in mouse models (40) (Fig. 3). Additionally, external CpG oligodeoxynucleotide (ODN) adjuvants co-administered with an AAV SARS-CoV-2 vaccine expressing the receptor binding domain (RBD) have improved vaccine efficacy (67). Although AAV stimulates TLR9, adding a TLR4-stimulating peptide (the agonist RS09, designed to mimic lipopolysaccharide) to the antigen accelerated the onset of the humoral response, with RBD-specific antibodies being detectable within 2 weeks (68, 69) (Fig. 3).

Adjuvanting fusion molecules, such as complement C3d, have differential antigen-dependent effects (70). The C3d fusion is designed to mimic antigen activation of complement and covalent linkage and to facilitate antigen presentation by CD21-positive follicular dendritic cells. Fusing C3d to the merozoite surface protein 4/5 of *Plasmodium yoelii* decreased vaccine efficacy, whereas fusing C3d to a model antigen, hen egg lysozyme, improved the humoral response to that antigen roughly 50-fold. An alternative strategy to enhance antigen presentation is fusing the antigen to the MITD (Fig. 3). In a melanoma challenge mouse model, fusing the secreted antigen to the MITD reduced the tumor burden and prolonged survival (60, 71). In certain instances, the expression of the antigen itself may cause disease. To mitigate the potential oncogenic activity of an antigen such as the human papillomavirus (HPV) 16/18 E7 protein, groups have used fusions to HSP70 to prevent tumorigenesis after AAV-mediated target cell transduction (72, 73). Vaccination with this fusion peptide protected mice from primary, secondary, and even tertiary challenges with an HPV-induced cancer.

Another common means of adjuvanting AAV vaccines involves using cytokines or chemokines, with granulocyte-macrophage colony-stimulating factor (GM-CSF) being among those most frequently used (Fig. 3). GM-CSF has effectively improved vaccine responses when administered locally during vaccine administration in mice and non-human primates (NHPs) (74–76). Vaccination with an AAV vector encoding the HPV L1 protein co-administered with an Ad5 vector encoding GM-CSF increased the magnitude and duration of the humoral response against the L1 antigen in mice (77).

## PRECLINICAL DATA ON AAV VACCINES

Vaccines have had a long-standing role in infectious disease control and prevention, but are more recently garnering interest for cancer and genetic disorders, as well. There are more than 60 published papers on preclinical AAV vaccine studies, which show remarkable diversity in their design and implementation (Table 1). Despite employing a wide range of doses and delivery routes with diverse capsids and antigens, studies have predominantly used a strong ubiquitous promoter such as the CMV, CBA, or CAG promoter to increase antigen expression. We will now highlight how vaccine efficacy has been demonstrated using AAV vectors in various preclinical models.

**TABLE 1:** Overview of all published preclinical AAV vaccine studies^*a*^

Genre	Entry	Pathogen, genetic disorder, or disease	Vaccine antigen and modifications	AAV capsid	Promoter	Dose	Route of administration	Animal model	Challenge	Author
Cancer	1	Breast cancer	Her-2 mimitope; peptide display on Vp3 adjuvanted with AL(OH)3	AAV2	Not specified	10 µg	IM	BALB/c mice	Yes	(51)
	2	Breast cancer	Murine Neu antigen	AAV5 or AAV6	CMV	1e11 vg	Oral	BALB/c mice	Yes	(78)
	3	Cervical cancer	HPV E7 fused to HSP70	AAV2	CMV	5e10 vp	IM	C57BL/6 (H-2b) mice	Yes	(72)
	4	Colon cancer	Carcinoembryonic antigen (CEA); adjuvanted with GM-CSF	AAV1	CAG	2e11 vg	IM	C57BL/6 mice	Yes	(74)
	5	Colorectal and breast cancer	Secreted neoantigens and ovalbumin peptides; co-expresses PDL1 miRNA, PD-1 trap and TLR9 inhibitory sequences	AAV2	CMV	4× 1e8 vg weekly	IM	BALB/c mice	Yes	(61)
	6	HPV16/18 and related tumors	E7 oncoprotein; fused to HSP70	AAV1 and AAV2	CMV	2e11 vg	IM	C57BL/6 (H-2b) mice	Yes	(73)
	7	Melanoma	Ovalbumin	AAV3 and AAV6	CMV	3e11 vg	IM, IP	C57BL/6 mice	Yes	(79)
	8	Melanoma	Secreted ovalbumin peptide; VP2- and Vp3-fused ovalbumin peptides	AAV2	CMV	3e9 or 6e9 vg	IM	C57BL/6J mice	Yes	(80)
	9	Melanoma	Ovalbumin or truncated tyrosinase-related protein 1 (TRP-1) with A463M mutation and MHC localization tags; modified residue S663V; encapsulated in extracellular vesicle	AAV6	Not specified	1e7, 1e8, 1e9, 1e10 vg	IM	C57BL/6 and C57BL/6-Irf8+32-/- mice	Yes	(81)
	10	Melanoma (B16F10 cells)	Ovalbumin tumor-associated antigen; MHC localization tags	AAV6 S663V	CMV	1e10 vg	IM	C57BL/6 mice	Yes	(71)
	11	Melanoma (B16F10 cells)	Ovalbumin tumor-associated antigen; MHC localization tags	AAV6 S663V	CMV	1e10 vg per antigen (4e10 vg in combination)	IM	C57BL/6 mice	Yes	(60)
	12	Mesothelioma	TWIST1; co-administered with AAV PD-1 vector	AAV DJ	CMV	2e10 vg	IM	BALB/c mice	Yes	(82)
Genetic disorder	13	Alzheimer disease	Secretion amyloid-β peptide (Aβ)1-43 or Aβ1-21	AAV2	CMV	5e11 vg	IM	Amyloid precursor protein (APP) transgenic mice	N/A	(83)
	14	Alzheimer disease	Aβ 15 AA isoform	AAV2	CMV	5e10 vg	IM	BALB/c mice	N/A	(84)
	15	Alzheimer disease	Secretion Aβ1-43	AAV2	Not specified	5e11 vg	Oral	Amyloid precursor protein (APP) transgenic (Tg2576) mice	N/A	(85)
	16	Alzheimer disease	Secretion Aβ1-43 or Aβ1-21	AAV2	CMV	5e11 vg	IM	APPswe/PS1dE9 transgenic mice	N/A	(86)
	17	Alzheimer disease	Aβ−42 AA isoform; fused with cholera toxin B subunit (CB)	AAV2	CMV	5e10 vg	Oral, IN, or IM	PDAPPV717I transgenic C57BL/6 mice	N/A	(87)
	18	Stroke and epilepsy	NR1 subunit of the N-methyl-d-aspartate receptor	Not specified	CMV	1e9 vg	Oral	Rat	Yes	(88)
Infectious disease	19	Canine parvovirus (CPV)	Capsid antigen	AAV6	CMV	1e10 or 1e11 vg (two doses)	IP and IM	C57BL/6 mice	No	(89)
	19	Canine parvovirus (CPV)	Capsid antigen	AAV6	CMV	1e12 vg	IV	Beagles (canine)	Yes	(89)
	20	Chikungunya	CHIKV structural polyprotein gene	AAV1, AAV2, AAV5, AAV8, and AAV9	CMV	5e8, 5e9, 5e10 vg (one dose), or 5e10 vg (two doses)	IM	C57BL/6 mice	Yes	(90)
	21	Cytomegalovirus	CMV-pp65 and CMV-IE1	AAV2	CMV	1e8, 1e9, and 1e10 vg	IM	C57BL/6 HLA-A2 in a Kb background (HLA A*0201Kb, or A2Kb)	No	(91)
	22	Ebola	EBOV glycoprotein (GP); Ad5 GP prime	AAV-PO6	CMV	1e8, 1e9, and 1e10 vg	IM	B10.Br (MHC-2K) mice	Yes	(92)
	23	HCV	Non-structural protein (NS5B); co-express a mutant perforin	AAV8	Liver-specific	5e9 vg	IV	C57BL/6 mice	No	(93)
	24	HCV	NS3 and NS4	AAVrh32.33	CMV	5e11 vg	IM	C57BL/6 mice	No	(94)
	25	HCV	E2 glycoprotein	AAV8 and AAVrh32.33	CMV	1e11 vg	IM	BALB/c mice	Yes	(95)
	26	Herpes simplex virus type 2	HSV-2 glycoproteins B and D	AAV2	CMV	5e10 vg	IM	C57BL/6 mice	No	(96)
	27	HEV	ORF3	AAVMYO3	CMV	1e11 or 1e12 vg	IV	BALB/c mice	No	(97)
	28	HIV	GAG-1; SAdV24 GAG-1 boost	AAVrh32.33	CMV	3e9, 3e10, and 1e11 vg	IM	CB6F1 hybrid mice	No	(98)
	29	HIV	HIV-1 *env*, *tat,* and *rev* genes; co-administered with CMV IL-2 adjuvant	AAV1	CMV	1e10 or 1e11 vg	IM, IN, IP	BALB/c mice	No	(99)
	30	HIV	GAG-1	AAV2	CMV	1e10 or 1e11 vg	Oral	BALB/c mice	No	(100)
	31	HIV	Env gp160	AAV1, AAV2, AAV3, AAV4, AAV5, AAV7, AAV8	CMV	1e10 vg	IM	BALB/c mice	No	(101)
	32	HIV-1	GAG; AdC7 GAG boost	AAV1, AAV7, AAV8, and AAV9	CMV	1e9, 1e10, and 1e11 vg	IM	(BALB/c × C57BL/6)F1 hybrid mice	No	(102)
	33	HIV-1	Gag of HIV-1 clade B	AAV2, AAV7, and AAV8	CMV	3e10 or 1e11 vg	IM	*Macaca fascicularis* (crab-eating macaque)	No	(103)
	34	HIV-1	gag-PR-ΔRT, rev-gp160, RT-integrase (IN); added parts of reverse transcriptase and rev response elements	AAV2	CMV	1e13 DRP RT-IN and/or gag-PR-ΔRT with 3.2e12 rev-gp160 followed by 1e13 DRP RT-IN and/or gag-PR-ΔRT with 5e12 env (two doses)	IM	*Macaca mulatta* (rhesus macaque)	Yes	(104)
	35	HPV	L2; L2 epitope insertion into Vp3, with various adjuvants	AAV2 Vp3 only VLP	Not specified	10 µg	IM	C57Bl/6, BALB/c mice, and Zika hybrid rabbits	Yes	(52)
	36	HPV 16	Major capsid protein L1	AAV5 and AAV9	CMV	1e13 vg	IN	*Macaca mulatta* (rhesus macaque)	No	(105)
	37	HPV 16	Major capsid protein L1 (L1h)	AAV5	CMV	5e10 or 5e11 vg (up to three doses)	IN	C57BL/6 mice	Yes	(106)
	38	HPV 16	Major capsid protein L1; co-administered with GM-CSF vector	AAV2	CMV	5e10 vg	IM	BALB/c mice	No	(77)
	39	Infectious bursal disease virus (IBDV)	IBDV VP2	Avian AAV DA-1	CMV	1e9 vg	IM	Chickens	Yes	(107)
	40	Influenza	NP	AAVrh32.33	CMV	1e11 vg	IM	BALB/c mice	Yes	(98)
	41	Influenza	COBRA H1 hemagglutinin; CpG-enriched vector (internal adjuvant)	AAV8 and AAV9 (capsid screen)	CMV	1e6-1e12 vg	IM	BALB/c mice	Yes	)(40)
	41	Influenza	COBRA H1 hemagglutinin	AAV9	CMV	2e11 vg	IM	*Mustela furo* (ferret)	Yes	(40)
	42	Influenza	Hemagglutinin (HA)	AAV1	CMV	1e10 or 1e11 vg	IM, IN, IP	BALB/c mice	Yes	(99)
	43	Influenza and pneumonic plague	T4(HA4900)-AAV(VRCO1)	T4-AAV fusion	CMV	2e11 vg	IM	BALB/c mice	Yes	(108)
	44	Influenza H1N1	Hemagglutinin (HA) head or stalk, or nucleoprotein (NP)	AAV9	CMV	1e11 vg (three doses)	IN	C57BL/6 mice	Yes	(109)
	44	Influenza H1N1	Hemagglutinin (HA) head or stalk, or nucleoprotein (NP)	AAV9	CMV	7.5e12 vg	IN	*Mustela furo* (ferret)	Yes	(109)
	45	Influenza H1N1	Hemagglutinin (HA), nucleoprotein (NP), and matrix protein (M1)	AAV9	CMV	5e9 vg	IM	C57BL/6 mice	Yes	(110)
	46	Malaria	*Plasmodium yoelii* merozoite surface protein 4/5 (MSP4/5); AAV1 and AAV3 prime boost	AAV1 and AAV3	CMV	1e12 vg each	IM	BALB/c mice	Yes	(111)
	47	Malaria	*Plasmodium yoelii* merozoite surface protein 4/5 (MSP4/5); CTLA-4 Ig fusion with antigen	AAV1	CMV	1e9, 1e12, or 1e13 vg	IM	BALB/c mice	No	(112)
	48	Malaria	*Plasmodium falciparum* circumsporozoite protein (PfCSP); Ad5 priming and AAV boost	AAV8	CMV	1e11 vg	IM or IV	BALB/c mice	Yes	(113)
	49	*Mycobacterium tuberculosis*	Ag85A; Ag85A-Vp2 fusion	AAV2	CMV	1e10 vg	IM	BALB/c mice	No	(114)
	50	Nipah virus (NiV) and Hendra virus (HeV)	NiV G	AAV1, AAV8, and Rh32.33	CMV	2e10 or 1e11 vg	IM	BALB/c mice	Yes	(58)
	50	Nipah virus (NiV) and Hendra virus (HeV)	NiV G	AAV1, AAV8, and Rh32.33	CMV	6e11 vg	IM	Golden hamsters	Yes	(58)
	51	Rabies	G protein; t2a GM-CSF adjuvant	AAV2	CMV	2e6 FFU	IM	BALB/c mice	Yes	(75)
	52	Rabies	G protein	AAV9	CMV	2e11 vp	IM	BALB/c mice	Yes	(115)
	52	Rabies	G protein	AAV9	CMV	1e13 vp	IM	*Macaca fascicularis* (crab-eating macaque)	No	(115)
	53	Respiratory syncytial virus (RSV)	Wild-type F protein, pre-fusion F protein, G protein	AAV5	CMV or CAG	5e10, 1e11, 2e11 vg (IM) or 5e10, 1e11 vg (IN)	IM or IN	BALB/c mice	No	(116)
	54	SARS-CoV-2	Spike receptor-binding domain (RBD); CpG ODN adjuvant	AAV2	CBA	2e11 vg (one or two doses)	IM	BALB/c mice	No	(67)
	55	SARS-CoV-2	Full-length spike protein or receptor-binding domain (RBD)	AAV5	CBA	1e11 vg	IM or IN	BALB/c mice	No	(117)
	55	SARS-CoV-2	Full-length spike protein or receptor-binding domain (RBD)	AAV5	CBA	5e9, 1e10, 1e11, and 5e11 vg	IM	Wistar rats	No	(117)
	56	SARS-CoV-2	Secreted receptor-binding domain (sRBD); 1×, 2×, or 3× RBD trimer	AAV8	CAG	1e9, 1e10, and 1e11 vg	IM	BALB/cJ mice	No	(118)
	56	SARS-CoV-2	Secreted receptor-binding domain (sRBD); 3× RBD	AAV8	CAG	5e10 or 5e11 vg	IM	Beagle (canine)	No	(118)
	57	SARS-CoV-2	Full-length spike protein or secreted receptor-binding domain (sRBD)	AAVrh32.33	CMV	1e10 or 1e11 vg	IM	Diet-induced obese (DIO) C57BL/6 mice, aged C57BL/6 mice	No	(119)
	58	SARS-CoV-2	Secreted receptor-binding domain (sRBD)	AAV9	CAG	1e11 vg	IM	C57BL/6J mice	No	(120)
	58	SARS-CoV-2	Secreted receptor-binding domain (sRBD)	AAV9	CAG	1e10, 1e11, and 1e12 vg	IM	Macaca mulatta	No	(120)
	59	SARS-CoV-2	Spike receptor-binding domain (RBD); secreted-RBD fused to RS09 peptide	AAV6 S663V	CAG	1e11 vg	IM	BALB/c mice	No	(69)
	60	SARS-CoV-2	Full-length spike protein or secreted receptor-binding domain (sRBD)	AAVrh32.33	SV40 or CMV	1e10 or 1e11 vg	IM	BALB/c mice	Yes	(121)
	60	SARS-CoV-2	Full-length spike protein or secreted receptor-binding domain (sRBD)	AAVrh32.33	SV40 or CMV	1e12 vg	IM	*Macaca mulatta* (rhesus macaque)	Yes	(121)
	61	SARS-CoV-2	Full-length spike protein or secreted receptor-binding domain (sRBD)	AAV11	SV40, EFS, CMV comparison	2e9, 1e10, and 1e11 vg	IM	BALB/c mice	No	(59)
	61	SARS-CoV-2	Full-length spike protein or secreted receptor-binding domain (sRBD)	AAV11	mCMV	1e11 vg	IM	*Macaca mulatta* (rhesus macaque)	Yes	(59)
	62	SARS-CoV-2	Full-length spike protein	AAVie	CMV	1e13 vg	IM	*Macaca fascicularis* (crab-eating macaque)	No	(122)
	62	SARS-CoV-2	Full-length spike protein	AAVie	CMV	6e10-1e11 vg	IM/IV	BALB/c mice	No	(122)
Model	63	Model antigen	Carcinoembryonic antigen (CEA); transduced alongside IL-4, GM-CSF, and CpG ODN	AAV6	CMV	1e3 MOI	*Ex vivo* transduction of dendritic cells (DCs)	C57BL/6 mice	No	(123)
Antigen	64	Model antigen	OVA epitope (aa 323-39); VP3 insertion	AAV2	Not specified	1.7 U+ 12 capsid (three doses)	IM	BALB/c mice	No	(53)
	65	Model antigen	*Plasmodium yoelii* merozoite surface protein 4/5 (MSP4/5) and hen egg lysozyme (HEL); fusion to c3d	AAV1	CMV	1e9, 1e10, 1e11, and 1e12 vg	IM	BALB/c mice	Yes	(70)

^*a*^ All preclinical vaccine studies found in PubMed that used AAV as the platform are described here. Administration doses are expressed in vector genomes (vg), viral particles (vp), DNase-resistant particles (DRP), multiplicity of infection (MOI), fluorescence-forming units (FFU), or units (U). Administration routes are listed as intramuscular (IM), intranasal (IN), or intravenous (IV).

### AAV vaccines for infectious diseases

Vaccines are arguably the best line of defense against most infectious diseases and are critical for preventing pandemics and improving public health overall. Unfortunately, immunogenicity and efficacy can sometimes be lower than desired, leading to more recent endeavors to assess the utility of new platforms over the currently commercially available options. Studies of AAV vaccines in NHP models of SARS-CoV-2 infection have highlighted the durability of the humoral immune response (up to 598 and 616 days in two studies) (59, 120). Vaccination using an ancestral strain of SARS-CoV-2 resulted in the development of antibodies reactive to more modern variants. Antigen-specific T cells peaked around week 10 and were still detectable at 44 weeks post-vaccination. Memory B and T cells were boosted in response to vector re-administration with an evolutionarily distinct capsid (121).

Analysis of the humoral response in AAV influenza vaccine studies of mice and ferrets using HA as the expressed transgene has demonstrated the induction of FcγRI-, FcγRII-, FcγRIII-, and FcγRIV-reactive antibodies (109). In a similar mouse study with the same transgene, the vaccine vectors elicited antigen-specific CD4+ and CD8+ T cells, and the responses could be augmented by using an internal CpG adjuvant (40).

In matched viral challenge studies, AAV-vaccinated animals exhibit minimal or no signs of replication of the challenge virus. In one such study, NHPs received a single 1 × 10^12^-vg dose of SARS-CoV-2 vaccine and were challenged 9.5 weeks later with 1 × 10^5^ plaque-forming units of virus (121). Virus replication was detected in nasopharyngeal and tracheal swabs from the unvaccinated control NHPs, but not in those from their AAV-vaccinated counterparts. When the dose was decreased to 1 × 10^11^ vg, some viral replication was detected during the challenge; however, the level was significantly lower than in unvaccinated controls (59). Similarly, the influenza challenge in both mice and ferrets showed that our AAV-vaccinated animals had complete protection against morbidity and mortality, along with diminished viral replication. The challenge virus was not detected above background levels in mouse nasal washes or lung homogenates. Similarly, it was detected in sneezes of unvaccinated control ferrets, but not in AAV-vaccinated ferrets. Notably, our AAV vaccine overcame pre-existing immunity to the antigen in the ferret model to confer protection against future drifted influenza strains (40).

In a study of an AAV vaccine against HIV, NHPs had antigen-reactive T cells after vaccination (104). Tetramer staining revealed T-cell expansion after booster doses and challenges in some animals. Antigen-reactive antibodies were also detected, and serum titers increased in response to the boosters and challenges. Post-challenge, viral RNA was tracked in plasma. Vaccinated NHPs had consistently less viral RNA when compared to unvaccinated controls, and none (0/5) showed signs of viral replication in response to low-dose challenges, whereas 3/5 unvaccinated controls did so; however, vaccinated NHPs had detectable virus after high-dose challenges.

### AAV vaccines for cancer

Cancer vaccines are generally newer and less well-developed than infectious disease vaccines. They similarly train the host’s immune system to target and destroy cancer cells. Some are designed to prevent the cancer from occurring in the first place (i.e., Engerix-B against hepatitis B; Gardasil against HPV), while others can treat existing cancer (i.e., Bacillus Calmette-Guérin against bladder cancer). AAV vaccines have extended survival in several cancer studies. Antigens used in preclinical studies have included neoantigens, full-length oncoproteins, and viral genes. Personalized neoantigen vaccines are rising in popularity as they can target abnormal markers expressed only by the tumor instead of healthy, normal tissue. Likewise, platforms can deliver full-length oncoproteins to capture the entire protein of interest or smaller viral genes, to increase the specificity and minimize any off-target effects. Immune-competent mouse models have shown robust anti-tumor responses after vaccination (60, 61).

An AAV vaccine encoding the HPV16 and HPV18 E7 antigens was developed to protect against HPV-associated cancers (72, 73). To mitigate the risk of transgene-mediated oncogenesis, the E7 protein contained point mutations and was fused to HSP70 to impede the full-length protein’s oncogenicity. Vaccinated mice were challenged with 1 × 10^5^ TC-1 cells (HPV-transformed lung epithelial cells). All control mice succumbed to disease within 4 weeks, whereas AAV1- and AAV2-vaccinated mice had 100% survival after primary and secondary challenges. After a tertiary challenge, AAV1- and AAV2-vaccinated mice maintained 100% and 70% survival, respectively. AAV1-vaccinated mice had significantly decreased tumor burden and increased IFN-γ-producing T cells, as compared to AAV2-vaccinated or control mice. T-cell production of IFN-γ was detected 52 weeks post-vaccination. Cytokine production was specific to the E7 antigen, as vaccinated mice challenged with control B16 melanoma cells exhibited no survival advantage over controls.

AAV6-based vaccines using the ovalbumin antigen have protected against challenge with B16F10 melanoma cells (71). In this study, the AAV6 capsid contained point mutations to facilitate APC transduction, and the ovalbumin antigen was fused to an MITD. Ovalbumin-reactive CD4+ and CD8+ T cells were detected in vaccinated mice, and the frequency of effector memory precursor cells increased with the optimized vector. After the challenge, mice vaccinated with the optimized vector had a reduced tumor burden and prolonged survival, succumbing to disease due to antigen-negative tumor relapse only after antigen expression ended. In follow-up studies, a vaccine using the tumor-associated antigen tyrosinase-related protein 1 (TRP1) provided increased protection in the mouse melanoma model (60). An AAV6-TRP1 vaccine not only reduced metastases more than did vaccines based on other tumor-associated antigens but also reduced the tumor burden and extended survival. Benefits were maintained regardless of whether the vaccine was administered before or after challenge with B16F10 cells.

Secreted neo-antigens have improved survival in a mouse colon cancer model (61) (Fig. 3). The designed vector expressed a secretion tag fused to CD4+ and CD8+ T-cell epitopes separated by a linker containing a furin cleavage site. To prolong the production of the secreted antigen, the vector also contained TLR9 inhibitory peptides, a PD-L2 domain to prevent PD-1 expression, and a microRNA targeting PD-L1. Optimized vectors improved CD4+ and CD8+ T cell responses. Mice receiving the vaccine combined with radiotherapy had a reduced tumor burden and extended survival after challenges with CT26 or 4T1 cells. Vector modifications enabled four sequential low-dose 1 × 10^8^ vg administrations, allowing for redosing and resulting in a significant immune response. All mice vaccinated with an optimized vector survived the primary challenge, and 60% survived a rechallenge, whereas no control mice survived.

### AAV vaccines for genetic disorders

AAV vaccines for genetic disorders have predominantly targeted Alzheimer’s disease. Accumulation of β-amyloid (Aβ) plaques is a hallmark of Alzheimer’s disease progression. Vaccinating transgenic mice with AAV vectors expressing Aβ epitopes reduced plaque formation during aging (85). Interestingly, this effect did not correlate with immune cell infiltration or hemorrhagic symptoms, despite high titers of antibodies reactive to Aβ epitopes. Cognitive function improved after vaccination, as quantified by mouse exploration and multiple maze tests (87). Follow-up studies confirmed that plaque clearance is associated with upregulated autophagy and elevated complement c1q and c3 in the brain (86).

## CLINICAL DATA ON AAV VACCINES

The only clinical trials of an AAV-based vaccine in humans have been Phase I and Phase II studies for HIV using an AAV2 vector encoding the GAG, the protease, and part of the reverse transcriptase of HIV-1 subtype C under the control of the CMV promoter. The trials followed preclinical studies in NHPs that demonstrated vaccine efficacy in reducing the viral load after high-dose challenges with simian immunodeficiency virus and that detected elevated T cells by tetramer staining after booster doses (104). In the Phase I study, doses ranged from 3 × 10^9^ to 3 × 10^11^ DNase-resistant particles (DRPs), increasing in log increments. Some patients were HIV-seronegative, healthy adults and were not excluded because of pre-existing AAV2 neutralizing antibodies (nAbs). The vector was safe and well tolerated, and no serious adverse events (SAEs) were reported. A minority of patients experienced mild symptoms such as fever and headache. The frequency of patients with GAG-reactive T cells was highest in the group receiving the 3 × 10^11^ DRP dose. The Phase II study incorporated a 3 × 10^12^ DRP dose escalation and boosters. Increasing the dose from 3 × 10^11^ to 3 × 10^12^ DRPs resulted in the percentage of patients with GAG-reactive T cells increasing from 16% to 38%. Pre-existing nAbs to the AAV2 capsid did not alter the frequency of GAG-reactive T cells. Interestingly, boosting with the same serotype at 6 months increased responsiveness to 44%, whereas boosting at 12 months did not improve efficacy.

## ADVANTAGES OF AAV VECTORS FOR VACCINES

Although considered inherently less immunogenic than other viral vectors, AAV offers numerous advantages over the others (124). Recombinant AAVs can be gutted to leave minimal viral elements in the final product (i.e., only the ITRs or packaging signals and the viral capsid). In other viral vectors, one or two open reading frames are usually deleted to make space for the payload, leaving the rest of the viral genome intact. For example, adenoviral vectors commonly have antigen-encoding genes inserted in place of the E1/E3 reading frames, leaving multiple other collateral adenoviral antigen genes intact. In contrast, an AAV vaccine is arguably safer, as it encodes only one foreign antigen: the capsid. With modifications, vector genome persistence can result in durable antigen expression, which is believed to be related to vaccine efficacy (66).

One significant advantage of AAV vaccines is the longevity of the immune response. In SARS-CoV-2 vaccine studies in humans, mRNA platforms typically generate antibodies that wane below the limit of detection after 6 months despite the use of boosters; in contrast, AAV platforms extended the humoral response to the spike protein from 598 to 616 days after a single dose in NHPs (59, 120, 125). Similarly, the AAV vaccines induced robust expansion of polyfunctional T cells, as IFN-γ- and IL-4-producing antigen-specific T cells were detected by ELISPOT.

Current vaccine technology has proved effective in healthy adults; however, vaccines are known to be significantly less effective in several high-risk populations (e.g., people with comorbidities or obesity, the elderly, and pregnant individuals) (126–129). Only one published study investigated AAV vaccine efficacy in preclinical mouse models of high-risk populations (119). A comparable humoral response was demonstrated in diet-induced obese mice, as compared to healthy controls; however, efficacy was diminished in 2-year-old mice modeling advanced age. Although this finding is promising, more work is needed to evaluate AAV vaccine efficacy in high-risk populations.

Another benefit of AAV vectors is their relative ease of storage and transportation when compared with other platforms. They are stable long-term at 4°C with carriers and can even be stored briefly at room temperature with no apparent functional consequence. The vectors are even resistant to freeze-thaw cycles. In stark contrast, mRNA requires long-term storage at −80°C, thus complicating the handling and distribution of mRNA vaccines (121).

Like mRNA and other nucleic acid-based vaccines for infectious diseases, AAV is easily programmable in that the antigen can be replaced as the virus evolves or to protect against a new variant. The sequence does not influence purification or handling (both are specific to the capsid) in the same way that a protein vaccine might. Additionally, using the patient’s own cells to produce the antigen alleviates some issues related to protein folding, as observed with RSV subunit vaccines (130–132).

AAV has established a promising safety profile throughout gene therapy trials, with SAEs and patient deaths being observed only in very high-dose clinical trials. The doses that elicit these responses are several orders of magnitude higher than those used in preclinical vaccine studies, as well as the two clinical trials. The recommended doses for FDA-approved therapies are typically dosed by weight (e.g., 1.33 × 10^14^ vg/kg for delandistrogene moxeparvovec-rokl to treat Duchenne muscular dystrophy; 1.1 × 10^14^ vg/kg for onasemnogene abeparvovec-xioi to treat spinal muscular atrophy; 6 × 10^13^ vg/kg for valoctocogene roxaparvovec-rvox to treat hemophilia A; 2 × 10^13^ vg/kg for etranacogene dezaparvovec-drlb to treat hemophilia B). The AAV vaccine against HIV used in clinical trials had a high dose of 3 × 10^12^ DRPs/patient, whereas delandistrogene moxeparvovec-rokl has a maximum dose of 9 × 10^15^ vg. To put this into perspective, that is 900× the highest dose in any published AAV vaccine study.

## DISADVANTAGES OF AAV VECTORS FOR VACCINES

### Carrying capacity

AAV vectors have a limited carrying capacity of ~5 kb, including the packaging signals and other regulatory elements, leaving ≤4 kb for antigen cDNA and generally limiting the vector capacity to a single antigen. In comparison, traditional vaccines can contain viral antigens from multiple serotypes: influenza vaccines are commonly quadrivalent, and HPV vaccines are multivalent, as are the more recent SARS-CoV-2 vaccine iterations. Expressing multiple large antigen-encoding genes, such as that for the SARS-CoV-2 spike protein (3,819 bp), in AAV vectors would probably require multiple distinct products.

Several groups have identified means of overcoming this limited carrying capacity. In an influenza vaccination study, instead of encoding multiple H1 antigens, the vector used encoded a computationally optimized broadly reactive antigen (COBRA), a single protein encoding conserved epitopes from evolutionarily distinct influenza strains (40) (Fig. 3). This strategy used a single immunogen to generate antibody breadth against a range of influenza strains, a feat not easily achieved with a wild-type antigen. Other means of generating broadly reactive immune responses include generating cross-reactive T cells that recognize multiple strains and directing responses toward the conserved region of the hemagglutinin stalk instead of the head. Alternatively, neo-antigens or immunodominant domains of proteins can be expressed to generate immune reactivity (133) (Fig. 3). We have also used non-canonical means to achieve antigen expression (134, 135). Placing model antigens such as GFP outside P5 can generate GFP-reactive CD8+ T cells. P5 is an alternative replication initiation site, and sequences outside P5 are regularly detected in AAV preparations using the promoter.

### Manufacturing

Compared to most biologics, AAV vectors are relatively challenging to manufacture. AAV *Rep* genes are cytotoxic, impeding cell line generation, and most vectors are generated via transient transfection of HEK293 cells. Alternatively, recombinant adenoviral or herpes simplex virus vectors can be used to transduce producer cells with helper virus genes and initiate production. However, this requires large-scale plasmid production and a substantial amount of transfection reagent to generate an adenoviral vector before generating an AAV; afterward, the vectors require nuclease treatment and chromatography purification. Although this expensive process is deemed cost-effective for treating monogenetic disorders such as hemophilia, it remains to be seen whether it will be financially viable for broad vaccination efforts (136).

### Potential for toxicity

Most AAV serotypes are highly hepatotropic when administered intravenously, prompting concern about liver toxicity (137). Though transient liver toxicity has been observed following intravenous administration for gene therapy purposes, these concerns are lessened in a vaccination setting due to the local administration route and comparably low dose utilized. The common vaccine delivery routes (e.g., intramuscular and intranasal) have only led to some, albeit minimal, hepatic transduction. Engineered serotypes with limited potential to transduce hepatocytes exist, but they are not widely used in preclinical vaccination studies (Table 1). A few studies employing capsid screens have demonstrated that vaccines generated using engineered capsids do not outperform naturally occurring serotypes. Transgene expression has been detected in the livers of NHPs and mice after intramuscular or both intranasal and intramuscular vector administration, respectively (120, 138, 139). Regardless of the animal model or administration route, AAV is likely to be detected in the liver. Some capsids with single point mutations can reduce hepatotropism, but these have not yet been tested in a vaccine context (140, 141). However, preclinical vaccine studies have detected no concerning hepatotoxicity as measured by elevated alanine transaminase or aspartate transaminase after vector administration (40).

As a nucleic acid-based vector, there is an inherent risk of AAV integration and related insertional oncogenesis (142). Among the thousands of patients treated with AAV vectors, no patient has developed a cancer that has been attributed to the vector. Briefly, in the context of wild-type AAV infections, hepatocellular carcinomas have been linked to AAV integration, although this is associated with elements, such as the 3′ CARE element, that are excluded from modern recombinant AAVs. Insertional oncogenesis has occurred in high-dose neonatal mouse models, but other studies contradict these findings. A common insertion site falls in a region of the mouse *Rian* locus that is not homologous to the human genome. Similarly, hepatic clonal expansion in dogs was associated with increased circulating transgene levels but was determined to be non-malignant. The relevance of these findings to human outcomes is unclear, as the ITRs and vector genomes predominantly integrate into homologous regions, yet no similar outcome has been reported in NHP studies or human clinical trials. The remarkable divergence in transgene design across studies further complicates the predictive value of integration patterns. Although there is an established risk-benefit assessment for gene therapy approaches, similar calculations for a vaccine with other available technologies may differ.

### Unintended immune outcomes

AAV vector administration can potentially produce dysfunctional CD8+ T cells and recruit regulatory T cells (Tregs). In a trial of gene therapy for α1-antitrypsin deficiency, administering an AAV1 vector led to Treg infiltration of injected muscle that was not observed under control conditions (143). CD8+ T cells had high PD-1 expression and did not produce IFN-γ in response to capsid stimulation, but IFN-γ was produced in the presence of checkpoint blockade. These exhausted capsid-reactive T cells persisted for 5 years. Dysfunctional T cells were also observed in preclinical studies of AAV vaccines using the HIV-1 GAG antigen. Compared to the AdC68 GAG vector, the immunogenicity of AAV7 GAG vectors corresponded to lower IFN-γ and TNF-α production with minimal expansion after booster doses. However, booster doses for SARS-CoV-2 resulted in CD8+ T-cell expansion, thus suggesting that the dysfunctional T cells are an antigen-specific finding (121). In contrast to AdC68-vaccinated mice, AAV7-vaccinated mice could not efficiently clear the vaccinia virus after viral challenge, and T cells displayed exhaustion markers. The researchers attributed these T-cell defects to the prolonged antigen expression achieved with AAV vaccination (102). A similar study using the HIV-1 GAG antigen yielded similar conclusions (102). Some capsids appeared more efficient than others at generating GAG-reactive IFN-γ-producing T cells (102). However, these cells were still unresponsive to an AdC7 GAG boost (102). This was attributed to a lack of IL-2 production, effector memory, and central memory phenotypes (102). Although it was concluded in both studies that this T-cell exhaustion was probably due to prolonged antigen expression from AAV vaccines, as compared with adenoviral vaccines, both studies also recorded high IgG2a and IgG1 antibody titers (102). This finding may be unique to certain antigens, as it has only been reported in a small portion of published preclinical vaccine studies (102, 144).

Another concern regarding vaccine development is the potential for inducing tolerance. AAV vectors expressing immunodominant myelin oligodendrocyte glycoprotein epitopes have induced tolerance in mouse models of multiple sclerosis with diverse immunological backgrounds (133). Administering a liver-targeted vector prevented encephalitis and prolonged survival. These benefits were Treg-dependent and could overcome pre-existing immunity to the antigens. Studies investigating the mechanism of tolerance induction by using the ovalbumin model antigen found that tolerance induction is dose-dependent (145). Low-dose hepatic expression of ovalbumin resulted in a functional CD8+ T-cell response, whereas high-dose hepatic expression led to Treg generation. Knocking out FOXP3, IL-10, or Fas-L in mouse models impeded tolerance generation. Tolerance induction can also be augmented by using rapamycin nanoparticles and IL-2 muteins (146). These findings extend to larger animal models, including dogs and NHPs (147). While tolerance to the *trans* gene has been documented in cases of hemophilia (148–151) and multiple sclerosis (133, 152, 153) gene therapy, to the best of our knowledge, this phenomenon has yet to be documented in AAV vaccine studies.

### Anti-capsid immune response

AAV vaccines must also overcome AAV immune history. As defined by anti-capsid antibodies, pre-existing immunity to AAV is relatively widespread, with 10%–60% of people having serotype-specific antibodies against AAV (154). Although the number varies based on the capsid and the global region assessed, most people are seropositive for naturally occurring capsids. As the efficacy of systemic AAV administration is significantly hampered by pre-existing immunity, most clinical trials and FDA-approved therapeutics have exclusion criteria related to the patient’s pre-existing immune status. If pre-existing immunity impedes the efficacy of a vaccine, its widespread use may not be feasible. But, in the only published clinical study on this topic, it was concluded that pre-existing immunity does not impede T-cell responses against the transgene product. Preclinical studies designed to address this issue have yielded inconsistent results. Studies modeling pre-existing immunity by using passive transfer of plasma containing defined levels of nAbs have shown a clear reduction in vaccine efficacy (155). This decrease appears to be linked to the capsid, as AAV2 vaccines are more susceptible to lower concentrations of nAbs than are AAV7 or AAV8 vaccines. For example, giving mice 24 mg of human IgG followed by vaccination with an AAV8 vector resulted in more GAG-reactive CD8+ T cells than in mice receiving 0.08 mg of human IgG and an AAV2 vaccine. In this study, a nAb concentration-dependent reduction in GAG-reactive CD8+ T cells was partially rescued by increasing the vaccine dose. Follow-up studies by the same group found AAV1 to be even better at evading pre-existing nAbs after passive transfer (103).

These findings are mirrored by those of a subsequent study in which vector-specific antibodies were administered intramuscularly. Similar passive transfer of pooled human IgG to mice resulted in reduced luciferase signal after intramuscular injection of AAV8 (138). The muscle signal decreased, and the liver signal was ablated entirely at a high enough concentration. Rhesus macaques were given two vectors nearly 2 years apart to determine whether this finding extended to larger animal models. An AAV8-vector-specific antibody was administered intramuscularly, followed by a different vectorized antibody in AAV9. The macaques had detectable anti-AAV9 antibodies at the time of vector administration, and all but one exhibited strong transgene expression. However, this result may be irrelevant to vaccine work as the administered dose of 3 × 10^12^ vg/kg was far greater than that in any published vaccine study.

Attempts to model pre-existing immunity by administering an irrelevant vector before the AAV vaccine (69) demonstrated that AAV vaccines administered after a GFP vector using the same capsid could result in an anti-transgene response against a SARS-CoV-2 antigen. The caveat is that the mice were given AAV6 (wild type or 663V)-GFP 2 weeks before AAV6 vaccination, which may be insufficient time to mount a robust anti-capsid response.

Whereas the vector was delivered intramuscularly in most preclinical AAV vaccine studies, some demonstrated efficacy with intranasal administration: After intranasal administration of AAV5 or AAV9 vectors encoding α-1-antitrypsin and LacZ, stable gene expression of both cassettes was obtained (139). After AAV9 administration, it continued for 9 months, even in the presence of high nAb titers. Similarly, triple intranasal vector administrations in mice and ferrets boosted vaccine efficacy (109).

Aside from the clinical trial, the most clinically relevant study may be one of rhesus macaques with natural pre-existing immunity to AAV vectors, in which all the animals had anti-AAV9 reactive antibodies (120). The anti-AAV9 titer did not rise significantly after intramuscular vector administration, and despite the pre-existing antibodies, a durable humoral response against the SARS-CoV-2 RBD was observed. Anti-RBD titers were dose-dependent, with the strongest response being observed in the high-dose (1 × 10^12^ vg) group. Titers in the low-dose (1 × 10^10^ vg) group hovered around the limit of detection, but they increased in the medium-dose (1 × 10^11^ vg) group and continued to escalate in the high-dose group. High-dose antibodies were durable throughout the 598-day monitoring period.

Fortunately, many different capsids show strong efficacy in capsid comparisons (40). If annual vaccinations are necessary to protect against a disease, the capsid could be replaced to overcome pre-existing immunity. Similarly, individual capsid serotypes can be tailored to evade pre-existing humoral immunity by synthesizing novel, engineered AAV capsids that avoid cross-reactivity of the currently circulating antibodies (156). It is important to note that AAV vaccine efficacy was not impaired by pre-existing immunity to the capsid in the only human trial (157, 158).

## CONCLUSIONS AND FUTURE DIRECTIONS

We have highlighted the versatility of AAV, an ideal virus not only for gene therapy but also for use as a vaccine platform. In preclinical studies, AAV vaccines have proved efficacious against infectious diseases, genetic disorders, and cancers, and their advantages promise a clinical impact that warrants further investigation (62).

Although pre-existing AAV seropositivity remains an exclusion criterion for enrollment, emerging conditioning regimens are showing promise in preclinical models. These strategies are extensively reviewed elsewhere (102, 144–149). Preclinical studies in various animal models have demonstrated that AAV vaccines promise to maintain efficacy in the face of pre-existing immunity (139). The only AAV vaccine study in humans also found no evidence of anti-AAV2 antibodies affecting vaccine efficacy, suggesting better translational value (157). Ultimately, the route of administration is a critical component in the context of overcoming pre-existing immunity; the localized intramuscular or intranasal administration of the vaccine vectors may not only mitigate risks of adverse effects but also allow for enough transgene expression to mount a robust immune response.

Ultimately, it is imperative to devise innovative and creative ways to help mitigate the impact of ongoing infectious disease epidemics and rising cancer rates worldwide. Vaccines are one such strategy. Despite the many vaccine modalities available, portions of the population remain unprotected. Notably, high-risk individuals (e.g., people with obesity or comorbidities, the elderly, and those with immunocompromising conditions) commonly exhibit blunted immune responses and immunosenescence, resulting in reduced vaccine efficacy. Unfortunately, these populations are increasing worldwide, emphasizing the importance of finding better ways to protect them. Furthermore, patients at increased risk of cancer recurrence after their initial treatments to maintain them in remission may benefit from anti-cancer vaccines.

Collectively, the data reviewed in this article highlight the immense potential of AAV as a vaccine vector. The available preclinical data warrant further consideration and exploration of AAV vaccines to improve public health.
